# Danger of Bathing When Heated

**Published:** 1885-02

**Authors:** 


### DANGER OF BATHING WHEN HEATED.

The London *Lancet* gives the following timely warning of the dangerous risk incurred in bathing when heated and perspiring:

"A fine young fellow, a trooper in the 3d Dragoon Guards, then on the march from Edinburgh to Manchester, took advantage of the night's halt to have a dip in the Wear near that city. Being strong, and a good swimmer, he took an oar, at which he worked for some time in the sultry evening till he came to deep water, and in a suitable place took his plunge. That he was immediately seized with cramps is evident from the statements of his companions, who, alarmed at his cries, hastened to render assistance, but he had sunk before they reached him, and he never rose again. When the body was recovered, a considerable time afterward, it bore every evidence of the cause of the disaster. It was described as being "twisted"—that is contorted; while the vessels of the head, especially in their gorged condition, pointed to congestion, in fact, to stagnation, of the circulation! That this young officer lost his life by bathing when in an over-heated condition, is quite clear.

"It would be well if soldiers and civilians would remember the lesson conveyed in the classical case of Alexander, quoted by Dr. Jones from Quintus Curtius, viz.: 'It was in the middle of one of the hottest days of a burning summer that Alexander arrived on the banks of the Cyndus. The freshness and clearness of the water invited the king, covered with sweat and dust, to take a bath. He stripped himself of his clothes, and, his body all in a sweat, descended into the river. Hardly had he entered, when his limbs became suddenly stiff, the body pale, and vital heat seemed by degrees to abandon him. His officers received him almost expiring in their arms, and carried him senseless to his tent.'"

